# Deep mitochondrial divergence within a *Heliconius *butterfly species is not explained by cryptic speciation or endosymbiotic bacteria

**DOI:** 10.1186/1471-2148-11-358

**Published:** 2011-12-12

**Authors:** Astrid G Muñoz, Simon W Baxter, Mauricio Linares, Chris D Jiggins

**Affiliations:** 1 Instituto de Genética, Departamento de Ciencias Biologicas-Facultad de Ciencias, Universidad de los Andes, Carrera 1 No 18A - 70, P.O.Box 4976, Bogotá D.C., Colombia; 2 Department of Zoology, University of Cambridge, Downing Street, Cambridge CB2 3EJ, UK; 3 Facultad de Ciencias Naturales y Matemáticas, Universidad del Rosario, Carrera 24 No 63C -69, Bogotá D.C., Colombia

## Abstract

**Background:**

Cryptic population structure can be an indicator of incipient speciation or historical processes. We investigated a previously documented deep break in the mitochondrial haplotypes of *Heliconius erato chestertonii *to explore the possibility of cryptic speciation, and also the possible presence of endosymbiont bacteria that might drive mitochondrial population structure.

**Results:**

Among a sample of 315 individuals from 16 populations of western Colombia, two principal mtDNA clades were detected with 2.15% divergence and we confirmed this structure was weakly associated with geography. The first mtDNA clade included 87% of individuals from northern populations and was the sister group of *H. erato *members of Andes western, while the second clade contained most individuals from southern populations (78%), which shared haplotypes with an Ecuadorian race of *H*. *erato*. In contrast, analysis using AFLP markers showed *H. e. chestertonii *to be a genetically homogeneous species with no association between mitochondrial divergence and AFLP structure. The lack of congruence between molecular markers suggests that cryptic speciation is not a plausible explanation for the deep mitochondrial divergence in *H. e chestertonii*. We also carried out the first tests for the presence of endosymbiontic bacteria in *Heliconius*, and identified two distinct lineages of *Wolbachia *within *H. e. chestertonii*. However, neither of the principal mitochondrial clades of *H. e. chestertonii *was directly associated with the patterns of infection.

**Conclusions:**

We conclude that historical demographic processes are the most likely explanation for the high mitochondrial differentiation in *H. e. chestertonii*, perhaps due to gene flow between Cauca valley *H. e. chestertonii *and west Pacific slope populations of *H*. *erato*.

## Background

Sequences derived from the mitochondrial genome are commonly used both in species delimitation and historical phylogeography. For example, deep divergence in mitochondrial DNA sequences (mtDNA) between related individuals is often taken as evidence for the existence of cryptic species [1-4]. The discovery of cryptic species-level variation has important implications for characterising biodiversity and for studies of speciation. Nonetheless, it is now well recognised that inference of evolutionary processes and species boundaries from mitochondrial sequences alone can be problematic [5,6]. For example, similar patterns of divergence can be due to host-parasite interactions, whereby selection leads different molecular markers to show different histories [7]. Mitochondrial lineages may also be retained through admixture between divergent species or populations [8], or perhaps due to unusual population structures [9,10]. Indeed, in diverse tropical radiations, mtDNA 'barcoding' may perform rather poorly as a species identification tool [6]. It is therefore of general interest to pursue individual cases of deep mtDNA divergence in order to determine how often such divergence is indeed an indicator of cryptic species-level variation.

*Heliconius *butterflies are an excellent ecological and genetic system for studying speciation. These unpalatable butterflies are recognized for their diversity of wing color patterns associated with mimicry [11,12]. The classical example of this adaptive radiation occurs between the comimetic species *H. erato *and *H. melpomene*. These butterflies co-occur in Central and South America and show convergent changes in their color pattern. They are represented by more than 20 different geographic forms which are considered subspecies [13]. Neutral molecular markers show geographic structure among subspecies of *H. erato *and *H. melpomene*, and most named races fall within a particular geographic clade [13-16]. However, in *H. erato *two forms: *H. e. hydara *and *H. e. chestertonii *are polyphyletic in the mitochondrial phylogeny [13,16]. Individuals of *H. e. chestertonii *fall into two distinct mtDNA clades that show over 2% divergence, with no clear biogeographic explanation [17]. *H. e. chestertonii *is found in the western Colombian Andes on disturbed, dry habitats and forms a hybrid zone with the geographically closest subspecies: *H. e. venus*. Although *H. e. chestertonii *is a member of the erato clade, it has an unusual wing color pattern compared with the characteristic red/yellow/black pattern of *H. erato*. *H. e. chestertonii *has an iridescent and melanic forewing while the hindwing displays a broad yellow band. The co-mimic for this wing color pattern is *H. cydno weymeri f*. *gustavi*, a member of the *H. melpomene *clade.

Rapid evolution in the early stages of the speciation process is expected to lead to incongruence between morphological and molecular markers [14,18,19]. However, in *H. e. chestertonii *the deep divergence in the mitochondrial haplotypes is not readily explained through ancestral polymorphism or hybridization. Such mtDNA division has not been observed in any other *Heliconius *taxon to date. One possible explanation might be that structure is due to the presence of endosymbiotic bacteria [19-21]. *Wolbachia *are intracellular bacteria and infect numerous species of arthropods and nematodes [22]. Interactions between these microorganisms and their eukaryotic hosts often has consequences for host reproduction, leading in some cases to breaks between populations or species [23,24]. *Wolbachia *are inherited maternally, so their evolutionary fate is tightly linked to that of the mitochondrion [25]. Furthermore, hybridization between *H. e. chestertonii *and its nearby relative, *H*. *e. venus*, produces partially infertile eggs [26]. Hybrid sterility of this form can also be generated by endosymbionts where parental populations are infected by different strains.

However, an alternative hypothesis for the mtDNA break within the continuous distribution of *H. e. chestertonii *is cryptic reproductive isolation, unrelated to endosymbionts. Given the emphasis on wing color pattern in *Heliconius *speciation, a potential case of cryptic speciation would be of considerable interest. To investigate these possibilities we first extend previous mtDNA sampling to better document the distribution of mtDNA lineages. In order to test for cryptic speciation, we have then complemented these data with nuclear Amplified Fragment Length Polymorphisms (AFLP) markers, to provide a comparison with nuclear biparentally inherited markers. Finally, we have tested for a variety of endosymbiotic bacteria to investigate whether mtDNA structure could be a result of patterns of infection among populations.

## Results

### Mitochondrial relationships

**Figure 1:**
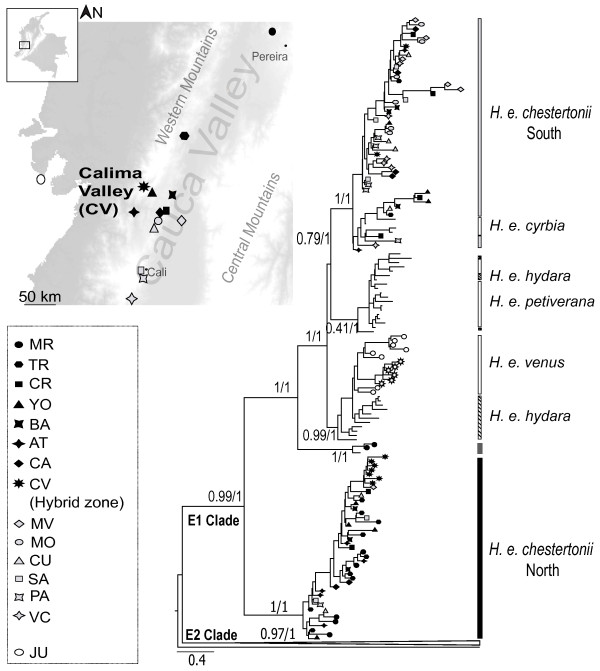
**Sampling sites of *Heliconius erato chestertonii *and *H. e*. *venus *and bayesian tree for the Clade E1 of *Heliconius erato***. Symbols correspond to population of origin for the individuals of *H. e. chestertonii*, south (gray) or north (black) and *H. e. venus *(white). The tree is based on mitochondrial genes of Cytochrome oxidase subunits I and II, leucine-tRNA and has the same topology as the Parsimony analysis. Posterior probability and bootstrap support are indicated on branches of the principal clusters. Individuals of the different subspecies are identified by bars (right).

Our expanded sampling of mitochondrial sequences confirms the deep divergence within *H. e. chestertonii*, with a similar topology obtained with either Bayesian or parsimony analyses (Figure 1). As expected, both taxa studied here are members of the E1 clade, containing *H. erato *species from Costa Rica, Panama, Colombia and west Ecuador [16]. *H. e. venus *forms a monophyletic group together with some members of *H. e. hydara*, while individuals of *H. e. chestertonii *showed a complex relationship with other subspecies and is a polyphyletic taxon. Three principal clades are observed. The first primarily includes individuals collected in populations north of the hybrid zone in Calima River Valley (Figure 1). These haplotypes form a sister group to the other subspecies of the western clade E1: *H. e. petiverana*, *H. e venus *and *H. e. hydara *(Figure 1). The second principal clade includes the west Ecuadorean race *H. e. cyrbia *and primarily individuals of *H. e. chestertonii *collected in southern populations (Figure 1). The third clade, with only three northern individuals, is sister to all races of *H. erato *in the western E1 clade and south clade of *H. e. chestertonii*. The different clades involving members of *H. e. chestertonii *has strong bootstrap support (100%) and a high posterior probability (1) (Figure 1). A haplotype network analysis revealed the same relationships among populations (Additional file 1). Three main star-like species/geographical clusters, *H. e. venus*, *H. e. chestertonii*-North and *H. e. chestertonii*-South. A topology test showed that monophyly of *H. e. chestertonii *could be strongly rejected (*P <*0.0001 Shimodaira-Hasegawa test).

Genetic analysis of mitochondrial DNA (mtDNA) within populations, showed that *H*. *e. chestertonii *is more polymorphic than *H. e. venus *(*θ_W _*= 0.01 and *θ_W _*= 0.003 respectively, Table 1). We then subdivided *H. e. chestertonii *to compare polymorphism within (1) northern and southern populations and (2) the two principal clades obtained in the phylogenetic tree. The northern and southern populations had similar levels of DNA polymorphism (*θ_W _*= 0.01 and 0.009 respectively) with no fixed difference between groups and 35 shared mutations (Table 1). Nonetheless, the comparison between clades showed 17 fixed differences and just three shared mutations (Table 1). The Analysis of Molecular Variance (AMOVA) showed that the maximum variability is within populations (63.65%) and only 18.22% of the total variability was due to geography, and a further 18.13% explained by species (groups in the AMOVA). When the species were analyzed separately, we found higher genetic structure among localities of *H. e. chestertonii *than between *H. e. venus *populations (*F_ST _*= 0.2775, *P *< 0.01; *F_ST _*= 0.1197, *P *> 0.5; Additional file 2).

**Table 1:** Description of mtDNA polymorphism among populations of *H. e. chestertonii*

Species/Population/Clade	(*S*)	(*h*)	(π)	(k)	(θw)
*H. e. venus*	11	6	0.0036	4.13	0.00339
*H. e. chestertonii*	59	29	0.0121	13.9	0.01028

Marsella (MR)	30	5	0.0136	15.6	0.01145
Trujillo (TR)	31	6	0.00959	11	0.00994
Yotoco (YO)	29	4	0.0127	14.6	0.01378
Buenos Aires (BA)	31	5	0.01516	17.4	0.01296
Carbonero (CR)	33	6	0.01417	16.26	0.01259
Caimital (CA)	26	4	0.0119	13.6	0.00992
Atuncela (AT)	27	5	0.01196	13.73	0.0103
Miravalle (MV)	33	6	0.01045	12	0.01259
Montañitas (MO)	4	4	0.00151	1.73	0.00153
La Cumbre (CU)	33	6	0.01341	15.4	0.01259
Saladito (SA)	24	4	0.01238	14.2	0.01004
Pance (PA)	24	3	0.00697	8	0.00916
Villa Colombia (VC)	3	3	0.00145	1.66	0.00114
Calima River valley (CV)	29	5	0.01096	12.59	0.00822
*H. e. chestertonii *(CV)	26	2	0.00647	7.42	0.00924
*H. e. venus *(CV)	20	3	0.00498	5.71	0.00711

North populations	52	21	0.01206	13.829	0.01017
South populations	42	16	0.0077	8.83	0.00889

North Clade	9	9	0.00125	1.43	0.00189
South Clade	23	18	0.00279	3.19	0.00456

The symbols in the table represent: number of segregating sites (*S*), number of haplotypes (*h*), nucleotide diversity per site (π), average number of differences between pair of sequences (*k*) and genetic diversity per site (*θw*).

### AFLP Analysis

Of the 327 loci examined, we found that 318 were polymorphic, representing 97% of loci. Genetic diversity statistics estimated from AFLP data showed that populations are differentiated within and among species (Additional file 3, *P <*0.001). Both *H. e*. *venus *and *H. e. chestertonii *had values of genetic diversity of 27.6%, while the hybrid zone populations (Calima River Valley) were more diverse than in any allopatric locality (*H. e. chestertonii *(CV) = 29% and *H. e. venus *(CV) = 30%).

**Figure 2:**
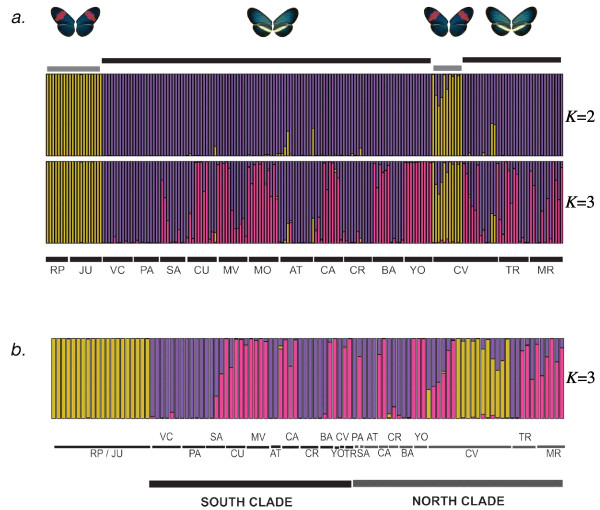
**Structure Analysis**. *a*. Graphical representation of results obtained from Structure. Upper black and gray bars represent the phenotype of each individual, *H. e. chestertonii *and *H. e. venus *respectively. The colours represent the Bayesian clusters when the analysis was carried out with *K *= 2 (upper, *L *= -20596.26) and *K *= 3 (lower, *L *= -20852.973) and correspond to *H. e. venus *(yelow) and *H. e. chestertonii *(violet and pink). The lower bars and letters show the population origin of individuals (for description of locations see Table 3). *b*. The results for *K *= 3 including only the individuals used in mtDNA analysis (*L *= -12353). Lower black and gray bars represent individuals in the southern and northern clades.

**Figure 3:**
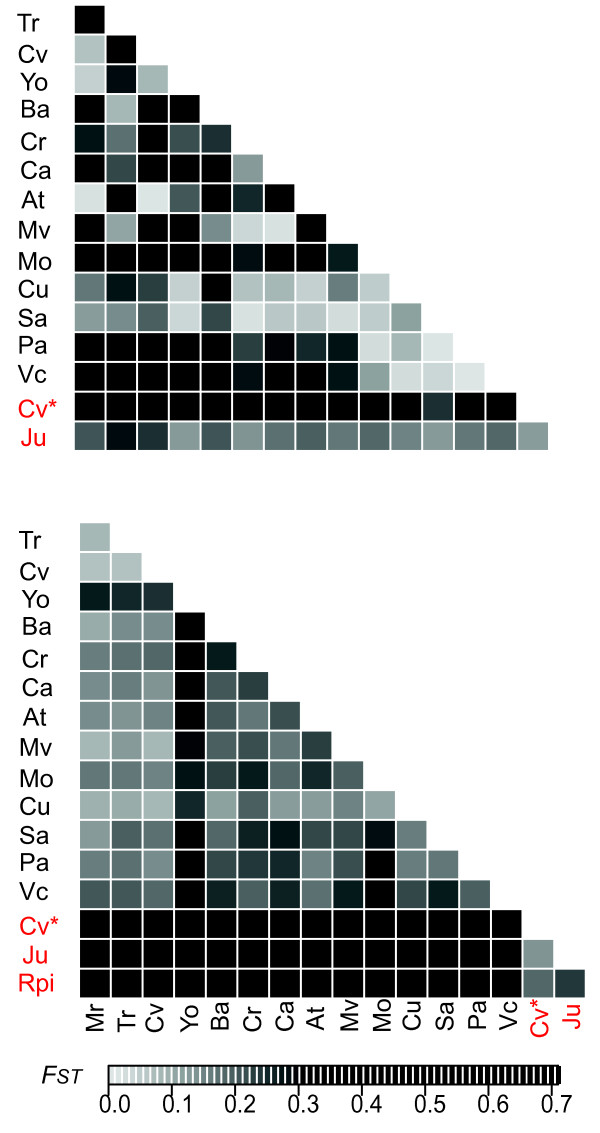
**Matrix of pairwise *F_ST_***. Upper and lower matrices show the *F_ST _*values for mtDNA and AFLP markers respectively, between each population. *H. e. chestertonii *(black) and *H. e. venus *(red) localities are provided in Table 3. In the Calima River Valley hybrid zone, individuals were separated by phenotype: *H. e. chestertonii *(CV) or *H. e. venus *(CV*).

The AFLP-based bayesian population assignment test identified two clusters which correspond to *H. e. chestertonii *and *H. e. venus *(Figure 2a, L = -20596.26). We compared the mean likelihood values for all runs with the statistic *ΔK *and confirmed that *K *= 2 is the best estimate for this data. Additionally, the PCA analysis gave a consistent result, with 56% of the variance in the data explained by differences between species (Additional file 4). In addition, all the populations used in this study showed significant genetic structure for AFLP markers (Figure 3 and Additional file 2). The AMOVA showed that 48% of the total variation in the AFLPs was explained within populations and the 42% among groups, which correspond to species in our analysis. When we excluded *H. e. venus *and repeated the AMOVA grouping data for northern and southern populations of *H. e. chestertonii*, only 9% of the variation was accounted for by geography. In order to test for congruent differentiation between AFLP and mtDNA markers, we ran a Structure analysis using only those individuals with data for mtDNA and assuming *K *= 3 (putatively representing *H. e. venus *and the two *H. e. chestertonii *clades). This analysis showed no evidence for any population structure associated with the mtDNA clades (Figure 2b).

### Interactions with endosymbionts

PCR analysis of 307 wild caught *H. e. chestertonii *did not identify any positive results for *Ricketsia *and *Spiroplasma *infection. All screens were run with a positive control of DNA from known infected individuals of other insects, so this failure is unlikely to be due to technical PCR error, although we cannot completely rule out the presence of a divergent strain of one of these bacterial taxa. In contrast, 7% of individuals screened (10 females/11males) were infected with *Wolbachia*. There are two major clades within *Wolbachia*, both of which are known to infect insects. Interestingly, the sequences derived from our samples fell into both clades, with 19 in clade-B and the remaining two in clade-A (Table 2). The presence of this endosymbiont is almost exclusive to *H. e. chestertonii*, with just one female *H. e. venus *from the hybrid zone infected. Most of these butterflies were collected in northern populations of *H. e*. *chestertonii *(85%). However not all individuals of the same population were infected and some localities did not show the presence of *Wolbachia*.

**Table 2:** Distribution of *Wolbachia *within populations of *H. e. chestertonii *and *H. e. venus*

Population (Individuals analized)	Negative diagnostic (Females/Males)	Clade-A (Females/Males)	Clade-B (Females/Males)
Marsella (33)	9/18		3/3
Trujillo (13)	2/10		0/1
Yotoco (4)	1/3		
Buenos Aires (22)	6/10	0/1	1/0
Carbonero (14)	6/6		1/1
Caimital (15)	0/9		3/3
Atucela (20)	4/16		
Miravalle (21)	8/12		0/1
Montañitas (20)	10/9		0/1
La cumbre (14)	4/10		
Saladito (10)	0/9		1/0
Pance (28)	11/17		
Villa Colombia (19)	6/13		
Calima River valley (44)	12/31	1*/0	
Juanchaco (29)	6/23		

The asterisk indicates the only infected individual of *H. e. venus*.

## Discussion

It is generally accepted that genetic differentiation between subpopulations can lead to the formation of new reproductively isolated species over time [27]. Phylogeographic analysis can be useful in identifying both cryptic species and incipient subpopulations on the way to becoming new species [2,3]. The analysis of this cryptic population structure can show how genetic, behavioral and ecological processes have acted during the earliest stages of speciation [27]. Studies that include independent sources of evidence, such as morphological comparisons, reproductive biology and phylogenetics, are necessary to understand the history of diverging lineages and to resolve species identification [28].

In this study, we first confirmed the unusual degree of mitochondrial divergence within *H. e. chestertonii*. Our analysis was based on a broad sampling of *H. e*. *chestertonii *which enabled the confirmation of two principal groups of mitochondrial haplotypes (Figure 1 and Additional file 1). Our broader sampling showed that these clades are not completely associated with the geographic distribution of the populations, as had been suspected previously (Figure 2b) [17]. The principal haplotype groups (Figure 1 and Additional file 1) were on average 2.15% divergent, similar to that estimated in previous studies [13,15,17]. This is considerably more divergent than within any other race of *H. erato*, apart from the trans-Andean biogeographic break which occurs within the distribution of the race *H. e. hydara *as described above. However, here there is no clear biogeographic context for this population structure. Nonetheless, the haplotypes do show some geographic structure, with 87% of northern individuals being Clade 1 and 78% of southern individuals Clade 2.

### Cryptic speciation

If cryptic speciation was occurring, whereby two species are considered as one based on wing pattern morphology, we would expect the mtDNA haplotypes to be associated with a detectable level of genetic differentiation at nuclear markers. Such cryptic species have been identified in a diversity of organisms such as bryophytes, fungi, elasmobranches and arthropods [1,4,27,29-32]. Within *Heliconius*, a case of cryptic speciation has been recently discovered in two closely related species: *H*. *timareta *and *H. melpomene *[33]. However, in *H. e. chestertonii *we have found that despite deep divergence in mtDNA between two principal clades (2.15%), the AFLP analysis shows *H. e. chestertonii *to be a genetically homogeneous species (Figure 2).

### Endosymbionts infection

Discordance between maternally inherited genetic markers and those transmitted biparentally, can often be explained by the spread of endosymbionts such as *Wolbachia*, *Spiroplasma*, *Rickettsia*, *Arsenophonus*, *Cardinium*, and others [34-37]. In most of the cases, these parasites are transmitted together with the mitochondrial genome through the egg cytoplasm, so associations over time can be detected when the mitochondrial genome is analyzed [38]. Some of these endosymbiont microorganisms can lead to reproductive alterations in their arthropod hosts and will lead to divergence between populations [19,39]. This is the first published study in which the presence of endosymbionts is tested in *Heliconius*, and we have identified two distinct lineages of *Wolbachia *within *H. e. chestertonii*. However, neither of the principal mitochondrial clades of *H. e. chestertonii *is directly associated with the infection (Table 2). We did not find evidence of presence of other endosymbionts in populations of *H. e. chestertonii*. We should add a caveat to our results, which is that our PCR assay might not have detected all possible infected individuals. Indeed, the stage of development of the host can lead to over or underestimates of the real density of *Wolbachia *within populations [22]. This study was limited to adult butterflies, and future analysis might include other stages such as egg, larvae and pupae. Nonetheless, the density of *Wolbachia *estimated within *H. e. chestertonii *populations (7%) would be considered "very low" according to a recent classification [40]. In the future it would be interesting to further investigate the phenotypic effects of this infection. It is also of course possible that another endosymbiont is present, which was not sampled with the PCR assays described here. However, for the moment there is no evidence that the mitochondrial structure in *H. e. chestertonii *is a result of endosymbiont infection. In summary, we have provided no evidence that mtDNA structure in *H. e*. *chestertonii *is due to either cryptic speciation or endosymbiotic bacteria. This leaves historical processes within the species as the most likely cause for the pattern.

### Historical processes

Climatic changes during glacial and interglacial periods can lead to contractions, expansions and fragmentations of populations [41-44]. The 2% divergence between lineages within *H. e. chestertonii *suggests divergence within approximately the last million years. Even without such vicariance, isolation by distance can lead to genetic structure within species. Intriguingly, recent work on *H. cydno *has shown that this species similarly has a marked genetic break in the center of its Cauca Valley range (Arias and Salazar, pers. comm.). In both species, southern Cauca populations are more closely related to subspecies on the Pacific coast than those in the North. A plausible scenario is that the Cauca Valley has been subject to a double colonization first from the central Andean valleys and subsequently from the Pacific populations in the west. Nonetheless, we are not aware of any geological evidence that would support this hypothesis.

## Conclusions

Our genetic analysis shows that northern and southern populations of *H. e*. *chestertonii *are genetically differentiated, but with only frequency differences in mtDNA clades, and no corresponding genetic structure at nuclear markers, despite the deep divergence (2.15%) between the two principal mtDNA clades. Our results support the assertion that mtDNA evidence alone should be used with caution in delimiting species boundaries. In this case, divergent haplotype groups within populations could not be explained by either cryptic speciation or endosymbiont infections.

## Methods

### Butterflies collections

Between 1997 and 2008, 315 specimens of *Heliconius erato chestertonii *and *H. e*. *venus *were collected at 15 different localities throughout southwest Colombia and one locality in Panama (Figure 1, Table 3). These two members of *H. erato *form a bimodal hybrid zone mantained by strong premating isolation in one of these Colombian localities [17,26]. The body of each butterfly was separated from the wings and preserved in DMSO 96% at -80°C, while wings were kept in glassine envelopes. Tissue and wings are stored in the Instituto de Genética, Universidad de los Andes, Colombia or in CDJs laboratory, University of Cambridge, UK (Table 3).

**Table 3:** Populations collected and individuals used in each analysis

Species	Locality (Code)	Latitude	Longitud			
				
		North	West	mtDNA	AFLPs	Endosymbionts
*H. e. chestertonii*	Marsella (MR)	4° 52.39'	75° 42.37'	5/1^¥^	12	33
	Trujillo (TR)	4° 13.30'	76° 20.30'	7/2^¥^	11	13
	Yotoco (YO)	3° 51.12'	76° 34.22'	4	4/6*	4
	Buenos Aires (BA)	3° 51.09'	76° 25.37'	6	11	18
	Carbonero (CR)	3° 44.53'	76° 28.56'	6	11	14
	Caimital (CA)	3° 44.12'	76° 30.41'	6	11	15
	Atuncela (AT)	3° 44.05'	76° 41.81'	6	12	20
	Miravalle (MV)	3° 41.22'	76° 21.18'	6	11	21
	Montañitas (MO)	3° 41.03'	76° 31.33'	1/5^¥^	11	20
	La Cumbre (CU)	3° 38.04'	76° 33.30'	6	11	14
	Saladito (SA)	3° 22.10'	76° 39.04'	4/2^¥^	9	10
	Pance (PA)	3° 19.27'	76° 38.11'	6	10	28
	Villa Colombia (VC)	3° 11.51'	76° 42.46'	6	11	19

*H. e. chestertonii/*Calima River Valley		3° 53.60'	76° 37.57'	2_5^¥^/5^¥^/2^¥^	11/11/2	27/16
*H. e. venus */hybrids	(CV)					

*H. e. venus*	Juanchaco (JU)	3° 56.22'	77° 22.08'	5^¥^	12	29
	Rio Piedras (RP)^†^	7° 63.62'	78° 18.97'		8*	-

**^¥^**Sequences available and deposited by Arias *et al*. 2008 [GenBank: EU707581- EU707607]; *Individuals of Butterfly genetics group collection (Chris Jiggins Laboratory, Cambridge UK); ^†^Darien Panama

### DNA Butterflies extraction

Genomic DNA was extracted from one-third of thorax or the end of the abdomen using DNeasy Blood & Tissue kit (Qiagen) following the manufacturer's tissue-extraction protocol. The thorax extractions were used for mitochondrial and AFLPs analysis and abdomen extractions for endosymbiont assays (Table 3).

### Sequencing and phylogenetic analysis of mitochondrial markers

At least six individuals of each population, three of each sex when possible (Table 3), were used to amplify a total of 1551 bp of a mitochondrial region, which covered the subunits I and II of Cytocrome Oxidase (CO I_II) and leucine-tRNA. We used published primers and PCR conditions from Beltran *et al*. 2002 for our amplifications. Subsequently, all the PCR products were purified for sequencing with Exonuclease I and Shrimp Alkaline Phosphatase enzymes (Fermentas) and sent to Macrogen Sequencing Service (Macrogen, Korea). Sequence editing was performed using Geneious Ver. 5.4 [45]. Sequences were aligned using Clustal W Ver. 2.0 [46] and checked for reading-frame errors in protein-coding regions with MacClade Ver. 4.08 [47]. Sequences were deposited in GenBank (Access numbers: JF912810-JF912880). To complement our analysis, we included publicly available sequences of *H. erato *[13,16,17] and sequences of *H. hecalesia *and *H. clysonimus *were used as outgroups (GenBank access numbers in Additional file 5). Phylogenetic analyses were performed using two different approaches: Maximum-Parsimony (MP) and Bayesian inference (BI).

Maximum-parsimony (MP) methods were implemented in PAUP 4.0b8 [48], using a heuristic search and tree bisection-reconnection (TBR) branch swapping option. A majority rule consensus tree was computed whenever multiple equally parsimonious trees were obtained. Parsimony bootstrap support values were estimated through 1000 bootstrap replicates. A Bayesian analysis was conducted with GTR + I + G nucleotide substitution model, which was the best-fit model obtained in JModel Test Ver. 0.1.1 [49] based on a Hierarchical Likelihood Ratio Test (HLRT). Bayesian inference (BI) was carried out using four simultaneous chains for ten million generations of Markov Chain Montecarlo (MCMC), sampling every 100 generations. The consensus tree and posterior probability of the nodes was estimated with Mr Bayes Ver. 3.1 [50,51].

### Topology test and population genetics analysis

The Shimodaira-Hasegawa (SH) log-likelihood test, as implemented in PAUP 4.0b8 [48,52], was used to test the monophyly of *H. e. chestertonii*. To test this *a priori *hypothesis, BI analysis was first performed using the same parameters as described earlier but this time by enforcing the monophyly of the complex as a topological constraint. The SH test was then used to compare trees obtained from both constrained and unconstrained analysis.

We described DNA polymorphism in populations of *H. e. chestertonii *and *H. e. venus *(Table 1) with DnaSP Ver. 5.10.01 [53]. The measures employed were: 1) number of segregating sites (*S*), 2) number of haplotypes (*h*), 3) average pairwise number of differences between sequences (*k*) and genetic diversity (*θ*). An analysis of molecular variance (AMOVA) and *F_ST _*values were obtained in ARLEQUIN Ver. 3.5 to determine genetic structure between populations from mtDNA markers [54]. To investigate relationships between populations a haplotype network was constructed using NETWORK Ver. 4.5.1.6 [55].

### AFLPs, cluster analysis and genetic distance

Genomic DNA from 185 individuals from all the populations, was used for DNA fingerprinting with AFLP markers (Table 3). The DNA quality was quantified by spectrophotometry and only samples with an A260/A280 ratio between 1.8 and 2.0 were used. For AFLP generation, we applied the general method of Vos *et al*. [56] with minor modifications. The AFLP^® ^Core Reagent Kit (Invitrogen) was used for the digestion of 125 ng of DNA for samples with *Eco*RI and *Mse*I restriction endonucleases and the ligation with *Eco*RI/*Mse*I adaptors, following the manufacturers protocol. The best primer combinations (high variation and fragments between 50 to 400 bp) for pre- and selective amplifications were selected by comparing the final sequencing of eight different primer mixtures for 48 individuals (Additional file 6). Sixteen samples chosen at random were run in duplicate during the screening process to ensure reproducibility of selected markers. As result, primers carrying a single selective nucleotide at the 3-end: *Eco*RI+A (5-GAC TGC GTA CCA ATT CA-3) and *Mse*I+C (5-GAT GAG TCC TGA GTA AC-3) were chosen for the pre-amplification cycle. The conditions in this first PCR were: denaturation at 94°C for 60s, followed by 20 cycles with denaturation at 94°C for 30s, annealing at 56°C for 60s and extension at 72°C for 120s. Subsequently, selective amplifications were performed using four pairs of primers that contained three selective bases and *Eco*RI primers were labelled with the fluorescent dyes: *Eco*RI (FAM)+ACA/*Mse*I+CGT, *Eco*RI (VIC)+ACC/*Mse*I+CGT, *Eco*RI (PET)+ACT/*Mse*I+CGT and *Eco*RI (NED)+ACG/*Mse*I+CAC. For these amplifications we applied a touchdown PCR of 12 cycles with denaturation at 94°C for 30s, an annealing temperature with 0.7°C stepwise reduction from 65°C to 56°C for 60s and extension at 72°C for 120s, after which 23 additional cycles were run with fixed annealing temperature of 56°C for 30s. AFLP reaction products were labelled with an internal size standard (GeneScan™-500 LIZ™) and run on an ABI 3730 automated DNA sequencer (Applied Biosystems). Fragment data were collected and analysed with GeneMapper Ver. 4.0 (Applied Biosystems). All bands were visually confirmed and only unambiguous loci between 50 and 400 bp were included in the analysis. Each individual genotype was manually assessed. A binary data matrix of presence (1) or absence (0) of bands of each size by sample was generated.

We used the presence/absence matrix of AFLP fragments to differentiate all individuals and populations using a Bayesian clustering algorithm implemented in the program Structure Ver. 2.3.3 [57,58]. The number of clusters (*K*) was determined by comparing the likelihood ratios for *K *values between 1 and 6. Previous runs including more than 6 clusters were done and the likelihood values were lower and are not shown in this analysis. Each likelihood value was estimated with runs that involved a burn-in of between 10^4 ^and 10^7 ^MCMC generations with 10 interactions. An admixture model under the assumption of Hardy-Weinberg equilibrium was implemented in the runs. The best number of clusters was confirmed with the *ad hoc *statistic Δ*K *[59]. Additionally we implemented a principal component analysis (PCA) using the software Genetix Ver. 4.05. [60] to confirm the results and variation of our data. Measures of heterozygosity and variation were estimated with AFLP-SURV Ver.1.0 [61] and with an AMOVA in Arlequin [54].

### Determination of the presence of endosymbionts

A total of 301 individuals (at least 10 from each population), were used to search for the presence of endosymbionts (Table 2 and 3). We used PCR to test for the presence of *Wolbachia*, *Ricketsia *and *Spiroplasma *in *H. e. chestertonii *and *H. e. venus*. Where the presence of *Wolbachia *was detected, additional PCR was carried out to determine the *Wolbachia *clade (A or B). Amplifications were carried out with specific primers that amplified the *wsp *gene of *Wolbachia *(A and B-clade) and 16S rDNA for the other two bacteria (Table 4). We designed three novel specific primers from our *Heliconius wsp *gene sequences in addition to those proposed by Zhou *et al*. 1998 (Table 4). Different combinations of these primers allowed us to determine the presence of *Wolbachia *(diagnostic combination: H81F/H691R), *Wolbachia *A-clade (H308F/H691R) and *Wolbachia *B-clade (H81F/wsp522R). Positive controls for these PCR reactions were DNA from known infected insects supplied by Emily Hornett (*Wolbachia*) and Francis Jiggins (*Ricketsia *and *Spiroplasma*) in the University of Cambridge, UK. We followed the amplification conditions as described previously [35,62-65].

**Table 4:** Primers for mtDNA and endosymbionts genes

Gene	Target species	Primers	Sequence (5'-3')	Reference
*COI*	*Heliconius*	Jerry	CAACATTATTTTGATTTTTTGG	Beltran *et al*. 2002
		Pat	TTCAATGCACTAATCTGCCATATTA	
tRNA-leu	*Heliconius*	George	TAGGATTAGCTGGAATACC	Beltran *et al*. 2002
and *COII*		Imelda	CATTAGAAGTAATTGCTAATTTAACTA	
*Wsp*	*Wolbachia*	H81F	AACTAGCTACTACGTTCGTTTGC	This study
		H681R	GCTACTCCAGCTTCTGCAC	
	A-clade	H308R	TTAAAGATGTAACATTTGACC	
	B-clade	wsp522R	ACCAGTTTTTGCTTGATA	Zhou *et al*. 1998
*16S*	*Ricketsia*	RSSUF	CGGCTTTCAAAACTACTAATCTA	von der Schulenburg
		RSSUR	GAAAGCATCTCTGCGATCCG	*et al*. 2001
*16S*	*Spiroplasma*	27f	GAGAGTTTGATCCTGGCTCAG	Hurst *et al*. 1999
		MGSO	TGCACCATCTGTCACTCTGTTAACCTC	Van kuppeveld *et al*.1992

## Authors' contributions

AGM obtained some specimens from Cauca Valley, carried out laboratory work, genetic and statistical analyses. SWB, ML and CDJ participated in the design and coordination of the study. AGM, SWB and CDJ drafted the manuscript. ML obtained most of the specimens from Cauca Valley used in this study. All authors read and approved the final manuscript

## Supplementary Material

Additional file 1 **Network of mtDNA haplotypes**. The median joining network of mtDNA haplotypes is congruent with the three clades in phylogenetic analysis (see Results). The colours represent *H. e. venus *(white), *H*. *e. chestertonii *samples of the north (black) and south of Cauca Valley (gray). Click here for file

Additional file 2 **Matrix with *F_ST _*values for mtDNA markers (lower triangle) and AFLP markers (upper triangle)**. Abbreviations correspond to localities. In the hybrid zone, individuals were separated by phenotype: *H. e. chestertonii *(CV) or *H. e. venus *(CV_V). Asterisk shows comparisons with significant values (*P *< 0.05). Click here for file

Additional file 3 **Genetic diversity from AFLP markers**. Estimates of total gene diversity (*H_T_*) and within group (*H_S_*). Index of fixation (*F_ST_*) was calculated grouping the populations within species (** *P <*0.01). Click here for file

Additional file 4 **Principal Components Analysis of AFLP markers**. The circles represent individuals of *H. e. chestertonii *and asterisks *H. e. venus*. Black circles showed individuals from south populations and gray circles those from the north. Click here for file

Additional file 5 **Sequences of species and subspecies from Genbank used in the mitochondrial analysis**. Click here for file

Additional file 6 **AFLPs combination primers**. Eight initial primer mixtures of selective amplifications. Click here for file
