# Supplementary figures and images for: Deep mitochondrial divergence within a Heliconius butterfly species is not explained by cryptic speciation or endosymbiotic bacteria
Source: BMC Evol Biol. 2011 Dec 12;11:358. doi: 10.1186/1471-2148-11-358 (PMC3287262; doi:10.1186/1471-2148-11-358)

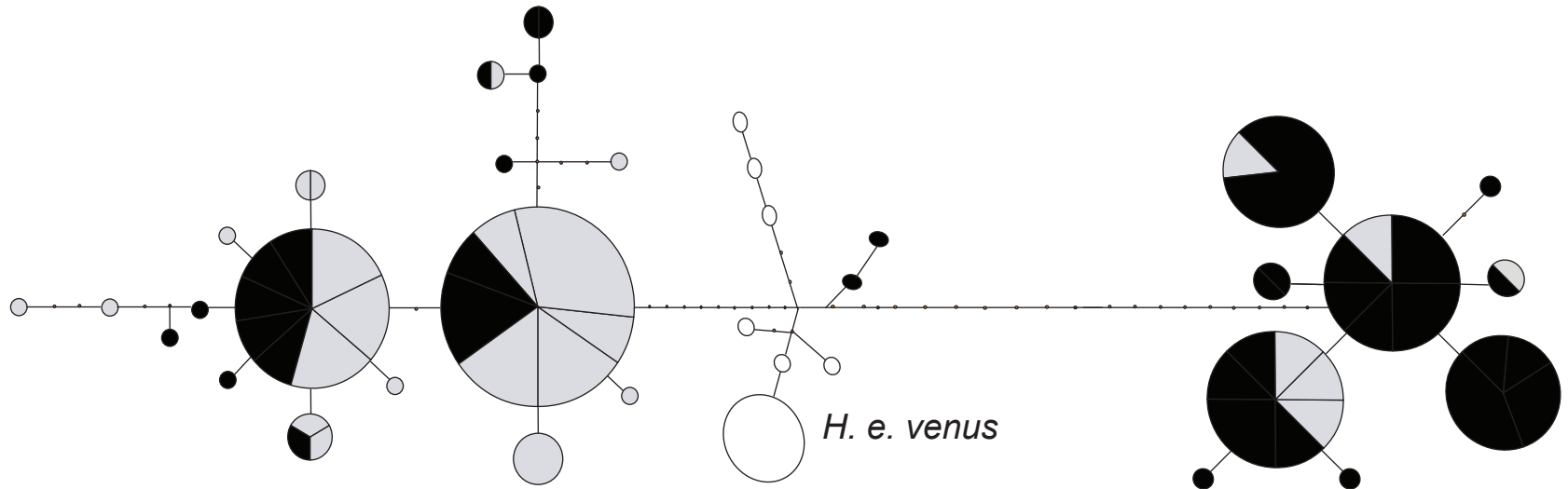

*H. e. chestertonii* - South

*H. e. chestertonii* - North

Supplement: Additional file 1 — Network of mtDNA haplotypes. The median joining network of mtDNA haplotypes is congruent with the three clades in phylogenetic analysis (see Results). The colours represent H. e. venus (white), H. e. chestertonii samples of the north (black) and south of Cauca Valley (gray). [file 1471-2148-11-358-S1.PDF]

PC2 (15%)

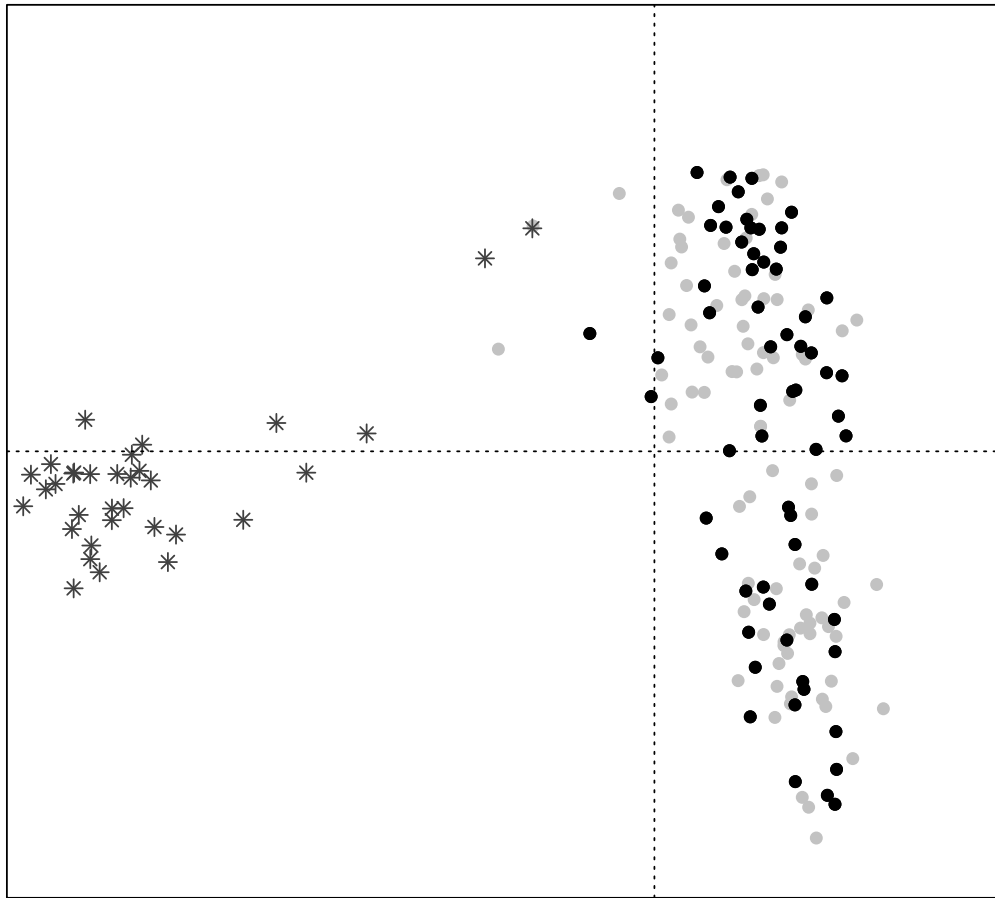

PC1 (56%)

Supplement: Additional file 4 — Principal Components Analysis of AFLP markers. The circles represent individuals of H. e. chestertonii and asterisks H. e. venus. Black circles showed individuals from south populations and gray circles those from the north. [file 1471-2148-11-358-S4.PDF]
