# Supplementary material for: Deep mitochondrial divergence within a Heliconius butterfly species is not explained by cryptic speciation or endosymbiotic bacteria
Source: BMC Evol Biol. 2011 Dec 12;11:358. doi: 10.1186/1471-2148-11-358 (PMC3287262; doi:10.1186/1471-2148-11-358)
Supplement: Additional file 2 — Matrix with FST values for mtDNA markers (lower triangle) and AFLP markers (upper triangle). Abbreviations correspond to localities. In the hybrid zone, individuals were separated by phenotype: H. e. chestertonii (CV) or H. e. venus (CV_V). Asterisk shows comparisons with significant values (P < 0.05). [file 1471-2148-11-358-S2.PDF]

	MR	TR	CV	YO	BA	CR	CA	AT	MV	MO	CU	SA	PA	VC	JU	CV_V	RPI
**MR**		0.072*	0.057*	0.243*	0.084*	0.145*	0.133*	0.132*	0.075*	0.150*	0.078*	0.119*	0.145*	0.177*	0.515*	0.379*	0.570*
**TR**	0.310*		0.059*	0.227*	0.133*	0.156*	0.142*	0.121*	0.119*	0.149*	0.087*	0.169*	0.159*	0.183*	0.484*	0.349*	0.532*
**CV**	0.063	0.350*		0.225*	0.133*	0.166*	0.121*	0.134*	0.075*	0.138*	0.074*	0.162*	0.133*	0.167*	0.496*	0.356*	0.549*
**YO**	-0.043	0.292*	-0.076		0.328*	0.363*	0.298*	0.330*	0.270*	0.255*	0.233*	0.398*	0.387*	0.395*	0.587*	0.466	0.655*
**BA**	0.415*	0.082	0.464*	0.365*		0.247*	0.179*	0.179*	0.174*	0.210*	0.104*	0.166*	0.203*	0.241*	0.511*	0.373*	0.564*
**CR**	0.275*	0.168*	0.309*	0.212	0.241*		0.211*	0.149*	0.196*	0.243*	0.174*	0.239*	0.213*	0.169*	0.509*	0.371*	0.571*
**CA**	0.349*	0.216*	0.381*	0.312	0.326*	-0.123		0.196*	0.153*	0.164*	0.108*	0.249*	0.233*	0.240*	0.471*	0.356*	0.517*
**AT**	0.019	0.352*	-0.006	-0.192	0.435*	0.251*	0.319*		0.212*	0.229*	0.107*	0.201*	0.134*	0.159*	0.461*	0.318*	0.506*
**MV**	0.361*	0.101	0.406*	0.311*	0.142	-0.029	0.014	0.344*		0.169*	0.137*	0.203*	0.193*	0.248*	0.532*	0.403*	0.594*
**MO**	0.564*	0.458*	0.642*	0.546*	0.448*	0.280*	0.367*	0.472*	0.269*		0.094*	0.259*	0.285*	0.301*	0.516*	0.393*	0.564*
**CU**	0.161	0.271*	0.230	0.036	0.353*	0.065	0.082	0.036	0.152	0.049		0.147*	0.148*	0.198*	0.456*	0.318*	0.495*
**SA**	0.119	0.137	0.189*	0.033	0.216*	0.017	0.059	0.057	0.027	0.046	-0.109		0.153*	0.248*	0.548*	0.406*	0.632*
**PA**	0.400*	0.425*	0.485*	0.315	0.442*	0.223*	0.294*	0.250	0.275*	0.024	-0.076	0.005		0.170*	0.517*	0.363*	0.587*
**VC**	0.553*	0.445*	0.633*	0.529*	0.439*	0.277*	0.360*	0.465*	0.270*	-0.113	0.024	0.033	-0.004		0.533*	0.380*	0.607*
**JU**	0.199	0.287*	0.244*	0.122	0.203	0.133	0.169	0.188	0.166	0.177	0.147	0.129	0.162	0.177		0.121*	0.166*
**CV_V**	0.614*	0.486*	0.700*	0.660*	0.471*	0.390*	0.532*	0.593*	0.410*	0.710*	0.322*	0.241*	0.550*	0.682*	0.120		0.214*
