# Supplementary material for: Deep mitochondrial divergence within a Heliconius butterfly species is not explained by cryptic speciation or endosymbiotic bacteria
Source: BMC Evol Biol. 2011 Dec 12;11:358. doi: 10.1186/1471-2148-11-358 (PMC3287262; doi:10.1186/1471-2148-11-358)
Supplement: Additional file 3 — Genetic diversity from AFLP markers. Estimates of total gene diversity (HT) and within group (HS). Index of fixation (FST) was calculated grouping the populations within species (** P <0.01). [file 1471-2148-11-358-S3.PDF]

Population or Specie	Number of polymorphic loci (%)	H_S	H_T	F_{ST}
Marsella	256 (82.1)	0.25823		
Trujillo	249 (79.8)	0.26329		
Calima River valley	261 (83.7)	0.27878		
Yotoco	245 (78.5)	0.16454		
Buenos Aires	224 (71.8)	0.23784		
Carbonero	223 (71.5)	0.22532		
Caimital	249 (79.8)	0.26006		
Atuncela	215 (68.9)	0.23945		
Miravalle	251 (80.4)	0.24556		
Montañitas	248 (79.5)	0.24426		
La Cumbre	251 (80.4)	0.27925		
Saladito	223 (71.5)	0.20027		
Pance	218 (69.9)	0.21884		
Villa Colombia	222 (71.2)	0.18020		
H. e. chestertonii		0.2354	0.2759	0.1468**
Calima River valley	255 (81.7)	0.30249		
Juanchaco	228 (73.1)	0.25874		
Rio Piedras	181 (58.0)	0.19082		
H. e. venus		0.2507	0.2766	0.0924**
All populations		0.2381	0.3116	0.2357**
