# Supplementary material for: Deep mitochondrial divergence within a Heliconius butterfly species is not explained by cryptic speciation or endosymbiotic bacteria
Source: BMC Evol Biol. 2011 Dec 12;11:358. doi: 10.1186/1471-2148-11-358 (PMC3287262; doi:10.1186/1471-2148-11-358)
Supplement: Additional file 5 — Sequences of species and subspecies from Genbank used in the mitochondrial analysis. [file 1471-2148-11-358-S5.PDF]

**Species and Subspecies**	**GenBank accession number**	**Number of Sequences**	**Cite**
H. e petiverana	*GU330020 - GU330034*	*15*	*Quek et al., 2010*
H. e. hydara	*GU330035 - GU330038, GU330046 - GU330050, GU330058 - GU330063, GU330104 - GU330107*	*19*	*Quek et al., 2010*
H. e. cyrbia	*GU330051 - GU330054*	*4*	*Quek et al., 2010*
H. himera	*GU330055 - GU330057*	*3*	*Quek et al., 2010*
H. e. phyllis	*GU330064 - GU330070*	*7*	*Quek et al., 2010*
H. e. lativitta	*GU330071 - GU330076*	*6*	*Quek et al., 2010*
H. e. etylus	*GU330077 - GU330086*	*10*	*Quek et al., 2010*
H. e. favorinus	*GU330087 - GU330090*	*4*	*Quek et al., 2010*
H. e. emma	*GU330091 - GU330095*	*5*	*Quek et al., 2010*
H. e. erato	*GU330096 - GU330103*	*8*	*Quek et al., 2010*
H. e. chestertonii	*GU330039 -GU330045*	*7*	*Quek et al., 2010*
H. e. chestertonii	*EU707581 - EU707584, EU707585, EU707587 - EU707589, EU707591-EU707596, EU707600,*	*15*	*Arias et al., 2010*
H. e. chestertonii	*U08580, U08565, U08568*	*3*	*Brower, A. V 1994*
H. e. venus	*EU707586, EU707590, EU707598 - EU707599, EU707602 -EU707607*	*10*	*Arias et al., 2008*
H. e. venus	*U08569- U08570*	*2*	*Brower, A. V 1994*
hybrid (chestertonii x venus)	*EU707601, EU707597*	*2*	*Arias et al., 2008*
H. hecalesia	*AY748069*	*1*	*Beltran et al., 2007*
H. clysonimus	*AY748068*	*1*	*Beltran et al., 2007*
