# Supplementary material for: Deep mitochondrial divergence within a Heliconius butterfly species is not explained by cryptic speciation or endosymbiotic bacteria
Source: BMC Evol Biol. 2011 Dec 12;11:358. doi: 10.1186/1471-2148-11-358 (PMC3287262; doi:10.1186/1471-2148-11-358)
Supplement: Additional file 6 — AFLPs combination primers. Eight initial primer mixtures of selective amplifications. [file 1471-2148-11-358-S6.PDF]

EcoRI	MseI
FAM-ACA	-CAC
FAM-ACA	-CGT
VIC-ACC	-CAC
VIC-ACC	-CGT
NED-ACG	-CAC
NED-ACG	-CGT
PET-ACT	-CAC
PET-ACT	-CGT
